# Viruses in Soil: A Review of Methods, Distribution and Ecological Functions

**DOI:** 10.3390/toxics14100858

**Published:** 2026-09-28

**Authors:** Fantong Song, Siqin Bao, Cheng Chen, Meiling Xu

**Affiliations:** 1 College of Desert Control Science and Engineering, Inner Monglia Agricultural University, Hohhot 010010, China; songft2026@163.com (F.S.); baosiqin@163.com (S.B.); 2 Key Laboratory of Environmental Health Impact Assessment of Emerging Contaminants of the Municipal of Ecology and Environment, Shanghai Academy of Environmental Sciences, Shanghai 200233, China; 3 College of Environmental Science and Engineering, Yangzhou University, Yangzhou 225127, China

**Keywords:** soil viruses, viral ecology, biogeochemical cycles, virus–host interactions, metagenomics

## Abstract

Viruses are the most abundant and active biological organisms in soil ecosystems. However, their spatial distribution and ecological functions remain inadequately addressed owing to a lack of systematic analysis and the complexity of the soil matrix. Therefore, this review systematically summarizes current knowledge of soil viruses through an examination of research methods, spatiotemporal distribution, and ecological functions. Our review indicates that current progress of soil viruses remains constrained by technological inconsistencies and difficulties in viral isolation techniques within complex soil matrices. Therefore, viral distribution analyses are largely limited to small-scale sections of agricultural land, forest, pastureland, and grassland. There is insufficient exploration of viral distribution across large-scale geographic areas and desert soil environments, as well as RNA virus diversity. The characteristics of soil viral communities are primarily influenced by environmental factors such as temperature, moisture, and pH. Overall, we conclude that soil viruses are pivotal drivers of microbial community dynamics and biogeochemical cycling, although our comprehensive understanding is severely bottlenecked by complex soil matrices and inconsistent viral isolation methodologies. Future research should focus on methodological innovations such as long-read sequencing and single-cell approaches to decipher virus–host interaction networks and their ecological roles across large-scale and extreme environments.

## 1. Introduction

Viruses are a unique group of acellular entities that entirely depend on host cellular machinery [1]. Morphologically, viruses exhibit extensive diversity, with particle diameters ranging from approximately 20 nm to over 500 nm [2]. The viral genome is highly compact and may consist of four major nucleic acid types: double-stranded DNA, single-stranded DNA, double-stranded RNA, and single-stranded RNA [3]. However, due to methodological limitations and the instability of RNA viruses, current viral research has primarily focused on bacteriophages and DNA viruses, while studies on RNA viruses and fungal viruses have been relatively scarce [4]. In recent years, the widespread use of meta-omics approaches has led to an increase in RNA virus research [5,6]. Viruses are the most abundant biological entities on Earth. There are approximately 4.80 × 10^31^ bacteriophages worldwide, and they are widely distributed across various ecosystems [7,8,9,10]. Of these, approximately 97% inhabit soil and sediment environments. The concentration of virus-like particles (VLPs) in soil environments can reach up to 10^10^ VLPs [11]. These observations suggest that viruses play an important role in shaping the microbial community structure and global biogeochemical cycles [12,13,14]. Viruses were used for therapeutic purposes as early as the 1940s. Currently, agricultural phage therapy is widely applied to eradicate pathogenic bacteria in soil–plant systems [15]. However, research on soil virus ecology lags by nearly a decade compared with studies on marine ecosystem viruses [16]. This delay is mainly attributed to the unique physicochemical and micro-environmental characteristics of soil. Soil is a heterogeneous system composed of solid, liquid, and gas phases, wherein the solid fraction generally accounts for half of the soil’s volume, with the remaining pore space filled with gas, liquid, or gas–liquid mixtures [3]. This unique microenvironment provides suitable ecological niches for viruses and other microorganisms, and constrains the life activities of soil microorganisms. In addition, soil viruses are highly infectious and potentially spread across trophic levels through the food chain, which may threaten animal health and pose public health risks.

In recent years, soil virology has drawn significant attention and substantial research progress has been accomplished through the widespread adoption of high-throughput sequencing technologies. However, our understanding of soil viral diversity, distribution patterns, and ecological functions remains limited owing to the unique nature of the soil environment, incompleteness of viral genome databases, and limitations of research methods [13].

This study evaluates recent knowledge on soil viruses through the following five topics: (1) soil virus research methodologies (including extraction, purification, and metaviromics approaches); (2) occurrence patterns and spatiotemporal distribution of viruses across different types of soil ecosystems; (3) environmental factors—such as moisture, pH, and organic matter—that drive the structure and diversity of soil viral communities; (4) regulatory roles of soil viruses in biogeochemical cycles; (5) integrated environmental effects and ecological functions of soil viruses, including the regulation of host microbial communities, mediation of horizontal gene transfer, and influence on ecosystems. This review aims to provide a comprehensive framework to address knowledge gaps in soil virus research and offers theoretical support for future applications in agricultural and environmental management.

## 2. Literature Retrieval

As of 16 November 2025, this study conducted a systematic literature search on viruses in soil ecosystems using two major databases: Web of Science and ScienceDirect. An initial broad search using the term “soil virus” retrieved 74,689 articles from the Web of Science and 74,591 articles from ScienceDirect. To refine the literature search, we further optimized our search strategy by combining the three keywords “soil viruses,” “viral ecology,” and “auxiliary metabolic genes.” This combined search yielded a total of 140 articles. No restrictions were set on the publication year during the search; the retrieved articles were primarily published between 2014 and 2026, though they also included some earlier classic studies. Subsequently, articles were selected for downstream analysis based on strict inclusion criteria, encompassing studies focused on: soil virology, virus–host interactions, viral ecology within soil ecosystems, viral detection methodologies, and their ecological functions.

## 3. Soil Virus Extraction and Analysis Methods

### 3.1. Soil Virus Extraction and Purification

The efficient elution and recovery of viral particles bound to soil matrices is a critical initial step in soil virological studies. For this purpose, four buffer systems are commonly employed: 10% potassium citrate, 10% beef extract, 250 mmol·L^−1^ glycine, and 10 mmol·L^−1^ sodium pyrophosphate [17,18,19,20,21]. Among these, 10% beef extract solution exhibits superior elution performance from a sludge matrix [22,23], while potassium citrate is effective from sandy and loamy soils [20]. Because eluents inevitably contain impurities and virus concentrations are typically below the levels needed for downstream analysis, additional concentration and purification steps are required [24]. Currently, the three most commonly used methods for the purification of viruses from extracts are tangential flow filtration (TFF), polyethylene glycol precipitation, and cesium chloride density gradient centrifugation [25]. Among these methods, TFF is the most widely used primarily because its large surface area allows the rapid passage of a high volume of filtrate, and it reduces the likelihood of clogging [25]. During viral nucleic acid extraction, it is critical to ensure that the virus is pure. Column filtration and epifluorescence microscopy (EFM) can assess viral purity. It is essential to confirm that the viral sample is free from contamination by other microbial cells [25].

### 3.2. Soil Virus Identification and Analysis

Common techniques used to quantify purified viral particles include transmission electron microscopy (TEM), EFM, and flow cytometry (FCM). These methods differ significantly in terms of operating costs, throughput, capture efficiency, and suitable applications [26,27]. A detailed comparison is provided in Table 1. Overall, EFM is the method of choice to assess soil viral abundance because it has lower operating costs, higher sample throughput, and greater capture efficiency compared with TEM. Viral counts obtained using FCM exhibit a good linear relationship with the counts generated by EFM and TEM, although the counts using FCM are typically higher [28] and can be up to 1400 times greater [21]. Therefore, FCM is particularly suitable for samples with low concentrations of viruses [28]. In recent years, fingerprinting technology has been gradually applied to viral ecology research. Among these, pulsed-field gel electrophoresis (PFGE) and random amplified polymorphic DNA-PCR (RAPD-PCR) are commonly used methods. PFGE uses agarose gels to separate viral DNA by genome size and distribution, and generate fingerprint profiles [29,30]. Combined analysis using PFGE and TEM shows that the DNA of most viruses is distributed across genomes ranging from 60 kb to 145 kb [31]. However, this method cannot distinguish genetic diversity within viral samples because different viruses may have genomes with identical or similar sizes [29]. While PFGE relies on genome size, RAPD-PCR amplifies viral DNA using random primers, and it can resolve the genetic diversity of viral communities at higher resolution [31,32,33]. Narr et al. optimized this method by combining RAPD-PCR with fluorescent markers (fluorescent RAPD-PCR; fRAPD), and capillary sequencing for rapid analysis [32].

However, the soil environment is highly heterogeneous, and viral genomes lack a universal marker gene analogous to the 16 S rRNA gene found in prokaryotes, making it difficult to gain in-depth insights into viral communities [25,34]. Metagenomics approaches, particularly metatranscriptomics, have emerged as mainstream tools in virology due to their culture-independent nature and capacity to simultaneously profile viral diversity and functional potential. For instance, Starr et al. applied metatranscriptomics to uncover high RNA viral diversity in soils, identifying fungi as their primary hosts [35]. This underscores the efficacy of metagenomics in deciphering complex soil viromes. Despite these advances, the detection of RNA viruses still faces numerous methodological bottlenecks: detection typically relies on RNA extraction, reverse transcription, PCR amplification, or metatranscriptomic sequencing, followed by hidden Markov model (HMM) searches using the RdRp marker gene for identification [35,36,37]. However, RNA is prone to degradation during processing, which may lead to underestimation of viral abundance [35]; viral transcript abundance is far lower than that of host rRNA, and the rRNA removal step may introduce additional bias [36]; furthermore, over 60% of environmental RNA virus sequences lack significant sequence matches in public databases, and taxonomic annotation is highly dependent on the completeness of the database [35,36]. These limitations underscore an urgent need for methodological refinement and the continual expansion of reference databases.

Consequently, several persistent challenges exist in the field: inefficient viral recovery from soil matrices [37], reliable discrimination between active and dormant viral states [38], and the identification of unknown viral hosts. The resolution of prophage dynamics, characterization of virus–host interaction networks, and elucidation of mechanisms that govern viral community regulation require long-read sequencing platforms and single-cell analytical approaches. Continued methodological innovation remains the primary engine of progress in soil virology, which underpins efforts to define the central ecological roles that viruses fulfill within terrestrial ecosystems.

**Table 1:** Methods used in viral research.

Research Methods	Advantages	Limitations	References
Metagenomics	No need to cultivate virus samples Not dependent on DNA sequences Analyzes the diversity and function of viral communities	Cannot determine viral ecological activity and has difficulty in the differentiation of viral viability states Over-reliance on the viral gene library	[2,39,40]
Fragment, Recruitment, Assembly, Purification (FRAP)	Efficiently integrates multi-source metagenomic data, highly stable	Dependence on the integrity of reference libraries, affected by the quality of original data	[11]
TEM	Observation of viral morphology	High cost, low sample throughput, and lower abundance estimates	[27]
EFM	Cheap, high sample throughput	Cannot observe virion structure	[3]
Flow Cytometer	High sensitivity, fast speed, and high throughput	Unable to provide viral morphology; complex operating procedures Expensive equipment with high maintenance costs	[28]
PFGE	Distinguishes viruses with different genome sizes and reveals compositional differences in viral communities	Cannot distinguish viruses with similar genomes	[30]
RAPD-PCR	Cost-effective and high-throughput analysis of viral communities across diverse soil environments	Template is highly sensitive to concentration, which presents amplification limitation and inadequate assessment of viral diversity	[29,41]
Fluorescent RAPD	Rapid large-scale analysis of viral communities	Highly dependent on primer selection and technical requirements	[32]

## 4. Occurrence and Distribution of Viruses in Soil

Although viruses are ubiquitous inhabitants of terrestrial soil environments, their community composition, diversity, and abundance vary considerably with land use type, soil physicochemical properties, and anthropogenic management regimes. Land use categories govern habitat quality, resource availability, intensity of biotic interactions, and the composition and function of resident biological communities, which collectively drive pronounced spatial heterogeneity in local biodiversity [42]. This review systematically compares viral abundance in agricultural lands, forests, grasslands/wetlands, and other terrestrial environments.

### 4.1. Agricultural Soil

Viral abundance in agricultural soils generally ranges from 10^7^ to 10^9^ g^−1^ dry weight, although considerable variation occurs across different regions [10,20,43,44]. For example, agricultural soils in Delaware contain 8.7 × 10^8^ to 1.1 × 10^9^ g^−1^ viruses (Table 2) [10], whereas arable rotation soils near Dundee show slightly higher values of 1.1 × 10^9^ to 1.2 × 10^9^ g^−1^ [43]. In contrast, viral abundances in the maize–wheat rotation system in southern China are notably lower (approximately 10^7^ to 10^8^ g^−1^) [45]. This disparity may be attributed to the region’s dominant red clay soils, which are characterized by low fertility and nutrient content. Among soil types, agricultural soils bear the strongest influence of human activity, which is clearly reflected in the virus-to-bacterium ratio (VBR). Cropland soils in Delaware exhibit exceptionally high VBR values of 460–3000 (approximately 460 under stricter harvesting practices) [10,44]. This elevated ratio arises from the suppression of bacterial communities under intensive agricultural management combined with the inherent difficulty of recovering soil bacteria rather than an elevated abundance of viruses. Furthermore, land management intensity is a non-negligible factor. Viral abundance in native tallgrass prairie soil is significantly higher than that in no-till canola and conventionally tilled winter wheat fields [46]. Moreover, conventionally tilled wheat soil and its adjacent prairie soil only share five rare viral operational taxonomic units (vOTUs), and prairie soil contains approximately ten times more vOTUs than tilled soil [46]. However, at a finer spatial scale, there are no statistically significant differences in viral density among the rhizosphere, rhizosheath, and bulk soil compartments within a single agricultural field [43].

**Table 2:** Soil viral abundance under different terrestrial ecosystems.

Soil Types	Location	Land Use	Purification and Analysis Methods	Target Viral Fraction	Abundance (g^−1^ dw)	Analytical Limitations	References
Agricultural	Delaware, USA	Corn	Physical dispersion EFM	Free extracellular viruses	8.7 × 10^8^–1.1 × 10^9^	Tailless, filamentous, and mycoviruses were overlooked	[10]
	Dundee, UK	Wheat	Physical dispersion TEM	Free viruses	1.1–1.2 × 10^9^	RNA viruses were overlooked	[43]
	Hunan, China	Winter wheat and summer corn annual rotation	Ultracentrifugation EFM TEM	Free extracellular viruses	1.31 × 10^8^	Fungal viruses and RNA viruses were overlooked	[45]
	Thuringia, Germany		Physical dispersion EFM fRAPD	Free extracellular viruses	8.1 × 10^8^	RNA viruses were overlooked	[32]
	Michigan, USA	Corn-soybean-wheat rotation	Nycodenz density gradient centrifugation EFM RAPD-PCR	Free viruses	1 × 10^8^	RNA viruses and mycoviruses were overlooked	[47]
	Oklahoma, UK	Canola (no-till)	Ultracentrifugation EFM Metagenomic	dsDNA viruses	1.01 × 10^9^	RNA and mycoviruses were overlooked	[46]
		Winter wheat (conventional till)			5.7 × 10^8^		
Forest	Delaware, USA		Physical dispersion EFM TEM	Free extracellular viruses	1.31–4.17 × 10^9^	Tailless, filamentous, and mycoviruses were overlooked;	[10]
	Thuringia, Germany		Physical dispersion EFM fRAPD	Free extracellular viruses	7.8 × 10^8^–1 × 10^9^	RNA viruses were overlooked	[32]
Pasture/Prairie	Thuringia, Germany		Physical dispersion EFM fRAPD	Free extracellular viruses	1.6 × 10^9^	RNA viruses were overlooked	[32]
	Oklahoma, UK		Physical dispersion EFM fRAPD	dsDNA viruses	1.66 × 10^9^	RNA and mycoviruses were overlooked	[46]
Desert			Physical dispersion EFM	Free extracellular viruses	2.2 × 10^3^–1.1 × 10^7^	Overlooked Viruses with Solubility and Intracellular Properties	[48]
Wetland	Delaware, USA		Nycodenz density gradient centrifugation EFM	Free vi ruses	8 × 10^8^–3.9 × 10^9^	Parasitic viruses were overlooked	[44]

### 4.2. Forest Soil

Among terrestrial soil types, forest soils exhibit the richest viral abundance. Forest soils from Michigan and Delaware harbor some of the highest viral abundances recorded in terrestrial ecosystems [10,47], which establishes them as hotspots for viral populations. Overall, viral abundance in these environments typically ranges from 10^8^ to 4.17 × 10^9^ g^−1^, which is higher than that in terrestrial ecosystems. In contrast to agricultural soils, forest soils maintain a relatively balanced VBR, which generally falls between 4.6 and 12 [10,43,44]. The capacity of forest soils to sustain such high viral abundances can be attributed to several factors. First, dense canopy cover reduces soil surface temperatures, which may prolong viral particle integrity and extend functional longevity [49]. Second, the low intensity of human disturbance in forest ecosystems fosters a stable physicochemical environment, which favors sustained viral persistence and proliferation.

### 4.3. Grassland or Pastureland

Compared with agricultural and forest ecosystems, virological data from prairie and pasture soils remain limited. It is estimated that the viral abundance in these systems generally reaches 10^9^ g^−1^ [32,46]. For example, soils from ranches in south-central Oklahoma (USA) contain approximately 1.6 × 10^9^ g^−1^ [32] and similar viral abundances are observed in Thuringia, Germany [46]. Despite this relatively narrow range, viral populations are consistently higher in prairie and pasture soils than those in cultivated agricultural soils. This difference is largely attributed to the greater organic matter and moisture content in grassland systems, as well as considerably lower levels of anthropogenic disturbance. Viral abundance in pasture soils is particularly high, which can be linked to the application of organic fertilizers.

### 4.4. Other Terrestrial Ecosystems

In addition to typical terrestrial habitats such as agricultural, forest, and prairie ecosystems, special habitats harbor unique soil viral communities, including wetlands, dunes, and deserts. Among these, wetlands are characterized by high viral abundance. Viral abundance in wetland soils exceeds the average level found in agricultural soils and typically reaches 10^8^–10^9^ g^−1^ [21,44]. The abundance peaks at 5 × 10^10^ g^−1^ when measured using flow cytometry [21]. Wetland sediments harbor a rich diversity of viral taxa with high overall abundance, and exhibit significant variation in viral community composition and abundance across different sites, with some locations showing extremely high levels of viral enrichment [50]. In contrast with wetlands, deserts have low viral abundance, with marked differences among deserts of different climatic types. In tropical deserts such as those in Saudi Arabia and North Africa, soil viral abundance ranges from 2.2 × 10^3^ to 1.1 × 10^7^ g^−1^ [48]. Meanwhile, the cold desert of McMurdo Dry Valleys in Antarctica contains approximately 10^8^ g^−1^, which exceeds the values observed in hot deserts. This suggests that temperature is the primary factor that distinguishes viral communities from cold and hot desert environments, while water availability is the main limiting factor for viral abundance in arid soils. In the Outer Banks region of North Carolina, USA, the viral density in dune sand is approximately 5 × 10^7^ g^−1^ dry weight [21], which is among the lowest of all soil types studied. This is largely attributed to the extremely low amount of organic matter and moisture in dune substrates [51,52].

Based on the above data, viral abundance in soil across different land-use types generally falls within the same order of magnitude (10^8^–10^9^ g^−1^), with no significant differences among agricultural, forest, pastureland, and wetland soils. Among these, the upper limit for forests and wetlands can reach 4 × 10^9^ g^−1^, which is slightly higher than that of agricultural soils. The viral abundance in pastures and prairies is concentrated around 1.6 × 10^9^ g^−1^, which places it at the higher end of this range. In contrast, viral abundance in desert soils ranges from 10^3^ to 10^7^ g^−1^. Notably, current data on soil viral abundance exhibit a pronounced geographic bias toward temperate agricultural and forest ecosystems, leaving arid deserts, tropical rainforests, and deep subsurface horizons substantially underrepresented. This disparity likely stems from the logistical feasibility of reproducible sampling in the former environments. Conversely, extreme ecosystems such as deserts present formidable methodological hurdles for viral extraction due to low organic matter content, extreme temperature fluctuations, and inherently lower viral biomass. Consequently, the higher viral abundances frequently reported in forest and wetland soils may not reflect the realistic viral distribution patterns. Rather, these observations are likely attributed to differential extraction efficiencies across distinct soil physicochemical matrices; for instance, high organic matter often facilitates viral elution, thereby artificially inflating measured abundances. Future initiatives must prioritize the standardization of extraction protocols and the expansion of global sampling coverage [10,25]. Importantly, extraction and counting methods play a decisive role in the determination of viral abundance values. For instance, abundance estimates derived from EFM and FCM can diverge by 350- to 1400-fold [45]. Similarly, TEM has been shown to underestimate phage populations by a factor of 8 to 40 [7].

## 5. Environmental Factors That Influence Soil Virus Communities

Temperature, moisture, pH, and anthropogenic activities are key drivers of viral dynamics in soil. At low temperatures, phages maintain a lysogenic lifestyle and coexist with their bacterial hosts, while high temperatures induce phage degradation and the lytic cycle. Dry conditions favor lysogeny, whereas increased moisture promotes phage release via lysis. An acidic pH reduces viral survival and stability, whereas neutral-to-natural pH supports high viral diversity. Human activities, including organic compost application and chemical pesticide use, lead to massive increases in phage abundance and enrichment of auxiliary metabolic genes (AMGs) in the soil viral community (Figure 1).

### 5.1. Soil Temperature

Temperature strongly affects soil virus inactivation. Temperature and soil adsorption are the two main factors that control viral persistence [53]. In saturated soil, poliovirus type 1 and coxsackievirus B1 are completely inactivated within 12 days at 37 °C; meanwhile, they remain infectious for at least 180 days at 4 °C [54]. The poliovirus inactivation rate constants (k) are 0.064–0.095 log_10_ reduction per day at 15 °C and 0.133–0.154 log_10_ reduction per day at 27 °C [55]. At 40 °C, poliovirus and MS2 coliphage lose detectable infectivity within 1 day [55]. However, under simulated dry field conditions, viral inactivation tends to occur rapidly; the primary driving factor may not be temperature alone, but rather the synergistic effect of water loss and rising temperature. Furthermore, thermal tolerance varies markedly among viral taxa. The abundance of viruses in cold desert soils (2.3–6.4 × 10^8^ g^−1^) is significantly higher compared with that in hot desert soils (2.2 × 10^3^–1 × 10^7^ g^−1^) [56], which suggests that low temperature slows down virus inactivation and promotes long-term viral preservation. Beyond direct effects on survival, temperature also influences phage life strategy selection. At lower temperatures, bacteriophages preferentially enter the lysogenic cycle and establish a mutually beneficial relationship with host bacteria; however, warmer temperatures result in viruses entering the lytic cycle, which leads to host cell lysis and death [57]. Temperature indirectly controls phage life cycle transitions, and it shapes the composition and succession of soil bacterial communities. Moreover, it modulates the trophic coupling between viruses and their bacterial hosts, and these changes cascade into broader shifts in the soil microbial community structure. For example, the interaction intensity between T4-like phages and their bacterial hosts substantially declines as temperature increases [58]. These findings suggest that the latitudinal temperature gradient is the primary climatic driver of geographic variation in soil virus–bacterium interaction networks. As global warming continues to raise soil temperatures, viral persistence, replication strategies, and ecological functions will change. These shifts may cause profound impacts on global element cycles and overall ecosystem stability.

### 5.2. Soil Moisture

The composition and structure of microbial communities are primarily shaped by soil pore structure and soil moisture conditions [59,60]. These factors strongly influence the homeostasis and functionality of soil microbial ecosystems by controlling aqueous space properties, viral survival, and infection mechanisms. For instance, controlled laboratory experiments have revealed a non-linear relationship between soil moisture and viral survival. Viral persistence was observed to decline as moisture content approached 15%, but recovered at higher levels, indicating a critical zone of minimum viability between approximately 15% and 25% [53]. Viral infection methods and life cycle options are strongly regulated by soil moisture conditions. DNA viruses in moist soils predominantly initiate lytic replication cycles, accompanied by a conspicuous increase in transcriptional output [61]. Rewetting is a pivotal ecological trigger for life-cycle transitions. Approximately 46% of microbial cells are killed within 1 week of rewetting, which is attributable to lytic activation following the conversion of temperate phages from lysogenic to lytic replication under restored favorable conditions [62].

In summary, moisture regulates viral ecological behavior through a coherent biphasic pattern: lytic infection dominates under wet conditions and imposes robust top-down pressure on microbial assemblages, whereas lysogenic coexistence with hosts prevails during drought. Rewetting disrupts this steady state, precipitates prophage induction, and results in large-scale restructuring of microbial communities [63].

### 5.3. Soil pH

Soil pH is a key factor that shapes local and global prokaryotic communities [64,65,66,67]. On the one hand, pH directly affects viral viability and stability. For instance, Actinomycete phages maintain structural integrity within pH values of 5.5–9.0. Free phage particles exhibit poor survival in acidic soils, particularly at pH values below 6.0 [68]. Furthermore, soil texture influences viral survival and function through adsorption. Sykes and Williams found that the pH stability of actinomycete phages was significantly reduced (pH increased from 4.9 to 6.1) after adsorption to kaolinite, indicating that the microenvironment on clay surfaces can alter viral pH tolerance. This suggests that soil texture (clay type) can modulate the actual impact of environmental factors on viruses through adsorption [69]. On the other hand, soil pH indirectly affects soil viral communities by influencing host bacterial communities. Long-term pH gradient manipulation experiments spanning Europe and North America show the cross-continental generality of this regulatory mechanism: 99% of detected vOTUs display strict pH-niche partitioning, which occurs exclusively in acidic (pH 4.5) or neutral (pH 7.5) soils, and neutral environments support markedly greater viral diversity [70]. These findings underscore that a pH-driven structuring of viral communities constitutes a globally reproducible phenomenon rather than an artifact of localized conditions. Through direct physicochemical constraints and indirect host-mediated pathways, soil pH is a fundamental determinant of viral community spatial distribution and diversity [71].

### 5.4. Soil Management Practice

Anthropogenic activities are an important driver of the soil viral community [72,73,74]. Applying organic fertilizers such as livestock manure and sewage sludge significantly increases viral diversity and richness [75]. Viral abundance in cropland soils receiving organic amendments is four to five times higher than that in soils treated exclusively with mineral fertilizers [45]. Likewise, human-induced contamination profoundly alters soil viral ecology. Sulfidic soils exhibit elevated viral abundance, and the prevalence of viral AMGs in these environments is approximately 30-fold higher than those of background levels [76]. Similarly, soils contaminated with organochlorine pesticides harbor greater viral abundance and higher relative proportions of AMGs [77].

## 6. Regulatory Roles of Soil Viruses in Biogeochemical Cycles

The “viral shunt” explains nutrient regulation and cycling in soil systems. When viruses lyse host cells, they release organic matter containing carbon, nitrogen, and phosphorus. Surrounding microorganisms can take up these nutrients directly. The released nutrients re-enter the ecosystem as dissolved organic matter (DOM) and particulate organic matter, which contributes to global biogeochemical cycles [9,78,79]. Although viral shunting was first described in marine environments, Williamson et al. argued that it also applied to terrestrial environments [3]. Specifically, when viruses lyse their hosts, they release unstable carbon into the soil, increase bioavailable organic carbon, and influence microbial decomposition rates [3]. However, several key details remain to be clarified, including the turnover rates of microorganisms and viruses, as well as the physical constraint of the soil matrix structure on viral diffusion. Kuzjakov and Mason-Jones propose a mechanism that links viral lysis to plant nutrition. Plant roots release labile carbon (including DOM) into the rhizosphere, which stimulates microbial growth and nitrogen mineralization in soil organic matter) [12]. Subsequently, when viruses lyse these microbial cells, the released nitrogen becomes available for plant uptake. This promotes root growth and development, which enhances carbon allocation to the rhizosphere, and establishes positive feedback (Figure 2).

### 6.1. Carbon Cycling

In the soil carbon cycle, organic carbon fixed by plants typically requires microbial decomposition to enter subsequent cycles; in contrast, organic carbon released through viral lysis bypasses higher trophic levels and returns directly to the microbial community to create a rapid microbial loop. This “viral shunting” mechanism alters the distribution pathways and turnover rate of carbon within the soil food web [3,78,80,81]. In marine ecosystems, quantitative estimates indicated that viral lysis accounted for 8–42% of bacterial carbon production and released 0.1–5.2 μg C L^−1^ d^−1^, with 6–26% of photosynthetically fixed carbon flowing through the viral shunt [78,81]. Furthermore, viral abundance significantly influenced soil carbon cycling. There was a reduction in respiratory carbon loss in coniferous forest and grassland soils with viral abundance increases [49]. Experimental soil microcosm studies have further shown that virus addition can increase cumulative respiration by 30% and significantly alter dissolved organic carbon loss [82]. Similarly, the organic carbon content in virus-treated samples from glucose-amended agricultural soils is twice that of the control group. Furthermore, higher soil viral abundance alters carbon and nutrient cycling in terrestrial ecosystems [82]. Beyond the direct release of nutrients during cell lysis, viruses regulate biogeochemical processes via AMGs. When expressed, phage-encoded AMGs can reprogram host metabolic pathways to influence biogeochemical cycles [83,84]. For example, a diverse set of viral-carried carbohydrate-active enzyme genes play a significant role in soil organic carbon (SOC) degradation and carbon flux regulation [85]. Furthermore, AMGs encoding glycosidases are widely distributed across multiple biogeographic regions, participate in the degradation of SOC, and promote carbon turnover [86].

In summary, viruses show significant potential to shape terrestrial carbon dynamics. However, existing quantitative estimates are largely derived from marine ecosystems, revealing a critical gap in empirical data for soil carbon fluxes. Because current soil virology relies on correlative data and microcosm studies that may not fully represent realistic conditions.

### 6.2. Nitrogen Cycling

Microbial communities are the primary drivers of soil carbon and nitrogen biogeochemical transformations [87,88,89]. A significant amount of soil nitrogen is sequestered in microbial necrotic matter [90]. Therefore, phage lysis of host cells plays a critical role in the stabilization of nitrogen in soil [91]. Soil microcosm experiments show that microbial lysis and predation significantly promote soil nitrogen mineralization and plant nitrogen uptake, which provides a theoretical basis for viral regulation of the nitrogen cycle [92,93]. A comparative analysis of China’s three major rice cropping systems shows that soil phages significantly reduce nitrogen fixation capacity by lysing nitrogen-fixing host bacteria, and influence nitrogen cycling [94]. Root-knot nematodes may disrupt the legume-rhizobium symbiotic system and trigger a chain reaction in the soil nitrogen cycle [3]. In this mutualistic symbiosis, rhizobia use carbon from the host plant’s photosynthesis to convert atmospheric N_2_ into NH_4_^+^, and provide bioavailable nitrogen.

### 6.3. Phosphorus Cycling

Typically, under 6% of total soil phosphorus is present as soluble orthophosphate [95]. The remaining phosphorus is largely unavailable and exists as phosphorus adsorbed onto positively charged mineral surfaces, or precipitates with metal ions into insoluble salts [96]. In soil environments, phosphorus mainly occurs as organic phosphorus (accounting for 30–80% of total soil phosphorus, with inositol phosphates representing more than 50% of this fraction) and inorganic phosphorus [95]. Metagenomic and metatranscriptomic analyses indicate that virus-encoded AMGs associated with phosphorus metabolism (such as genes encoding alkaline phosphatase or those involved in phosphate transport) are upregulated to promote host phosphorus uptake and the dephosphorylation of organic phosphorus [95]. However, direct experimental evidence demonstrating that these virus-encoded AMGs make a functional contribution to soil phosphorus cycling remains limited; current conclusions are primarily inferred from gene composition and transcriptional activity. Upon completing replication and lysing host cells, phages release intracellular phosphorus into the soil matrix as bioavailable phosphates—a process fundamentally driven by the viral shunt. The potential scale of this phosphorus flux is substantial: microbial biomass phosphorus ranges from 8 g P m^−2^ in deserts to 70 g P m^−2^ in boreal forests, with croplands averaging approximately 12 g P m^−2^ [97]. Furthermore, phosphate-solubilizing microorganisms actively drive this cycle, capable of increasing available phosphorus by 2.1- to 4.4-fold within 30 days, yielding a net gain of approximately 38 mg kg^−1^ [95]. When plants and animals die, their remains are decomposed and mineralized by microorganisms, and some phosphates are passed down the food chain to herbivorous animals. When plants and animals die, their remains are decomposed and mineralized by microorganisms. Subsequently, phosphorus returns to the soil microbial community, which initiates a new cycle [95]. This virus-mediated phosphorus cycling mechanism accelerates biogeochemical cycling of phosphorus in ecosystems and the rate of nutrient turnover.

## 7. The Interaction Between Viruses and Other Microbiota

### 7.1. Interactions Between Soil Viruses and Host Bacteria

Phages primarily mediate their interactions with host bacteria through two life strategies: lysis and lysogeny [97,98]. In the lytic strategy, a phage infects a host, utilizes host intracellular resources for replication, eventually lyses the host cell, and releases progeny phages [99,100]. The lysogenic strategy is prevalent among temperate phages whose genomes integrate into the host bacterial chromosome and replicate synchronously with the host genome as prophages [99,100]. When the host encounters environmental stresses such as dehydration or DNA damage, prophages can be induced to enter the lytic cycle [61,101]. These two survival strategies shape distinct virus–host regulatory patterns. The lytic strategy conforms to the “kill the winner” theory, which maintains the structural homeostasis of soil bacterial communities through a suppression of the overgrowth of dominant bacterial populations [102]. In contrast, the lysogenic strategy follows the “piggyback-the-winner” framework, whereby phages exploit the host for replication while conferring superinfection immunity to the host [103,104,105]. Furthermore, soil microenvironmental factors such as soil moisture and porosity modulate the life strategy selection of phages to regulate the cohabitation of bacteriophages and host bacteria [3,106]. The combination of tea extract and ferrous sulfate as an anti-phage reagent reduces soil phage abundance by one order of magnitude, while increasing microbial cell counts by approximately 91% within a few days [107]. These results suggest that a short-term reduction in phage abundance can rapidly unleash the metabolic capacity of the host microbial community. Furthermore, phage-encoded AMGs encode key enzymes involved in core host metabolic pathways, including carbon, nitrogen, and sulfur cycles. During infection, these genes modulate and enhance host metabolic activity, which provides sufficient material and energy resources for phage replication [108,109]. There is significant enrichment of viral AMGs associated with pesticide degradation in organochlorine-contaminated soil, which indicates that phage-carried AMGs improve the host’s adaptability to exogenous pollutants [77]. Under persistent viral predation pressure, host bacteria have evolved complex antiviral defense systems. Phages and their host bacteria maintain close coevolutionary interactions, which are commonly described as an “evolutionary arms race.” Bacteria can simultaneously employ the clustered regularly interspaced short palindromic repeats (CRISPR)-Cas adaptive immune system and restriction-modification (R-M) system. These two defense pathways act synergistically to cleave invading phage DNA and substantially enhance bacterial resistance to phages. Even when phage DNA undergoes methylation, it cannot evade recognition and cleavage by the CRISPR-Cas system. Under this persistent selective pressure from the host CRISPR system, phages can evade immune detection through the accumulation of key single-nucleotide mutations in spacer sequences or PAM regions. This forces bacterial populations to rapidly update their CRISPR spacer sequences and expand population immune diversity. Phages evolve diverse strategies, while bacteria develop novel resistance mechanisms [110,111]. Such mutual adaptation drives the rapid coevolution of both sides [112,113]. Soil microscopic studies demonstrate that bacteria exposed to phages exhibit greater resistance than control groups, whereas phages evolve to infect a broader range of bacterial hosts [113].

### 7.2. Virus-Mediated Horizontal Gene Transfer

Horizontal gene transfer is a key evolutionary mechanism that enables genetic exchange across species boundaries and helps microbes adapt to changing environments. It mainly occurs through three pathways: transformation, transduction, and conjugation (Figure 3) [114,115,116]. Phage-mediated transduction is either generalized or specialized, which differs in the manner that genetic material is transferred and in its origin. Generalized transduction occurs during the lytic cycle. When fragments of host DNA are mistakenly packaged into phage capsids during assembly, they can be randomly transferred to new hosts [116,117]. Specialized transduction is linked to the lysogenic cycle. After a temperate phage integrates into the host genome, incorrect excision during induction can transfer adjacent host genes along with the prophage DNA [116]. In addition to these classical mechanisms, recent studies show that lateral transduction is a highly efficient mode of gene transfer. This process enables the movement of extensive host DNA regions located near the prophage integration site, and its efficiencies exceed those of generalized transduction by several orders of magnitude [108]. As a result, lateral transduction significantly accelerates bacterial adaptive evolution.

Lysogeny is a central replication strategy of temperate phages that plays a pivotal role in virus-mediated gene transfer. Once integrated, prophage-encoded genes can be stably maintained and expressed within the host genome, which is known as lysogenic conversion [118,119]. This mechanism is important in pathogenic bacteria. For example, virulence factors such as diphtheria toxin, cholera toxin, Shiga toxin, and botulinum toxin are encoded by prophage-associated genes and enhance host pathogenicity through lysogenic conversion [118]. These observations highlight that phages are vectors of genetic exchange and key drivers of bacterial virulence evolution.

Spontaneous induction of prophages represents an additional pathway that facilitates gene dissemination. In the absence of external stress, a small subset of lysogenic cells can undergo induction, which leads to phage release. This process is often described as “altruistic,” and may provide a competitive advantage at the population level while promoting the spread of beneficial genes within microbial communities [114,119].

It is widely recognized that viruses are important reservoirs and vectors for the spread of ARGs in environmental systems and viral assemblages commonly harbor ARGs associated with diverse resistance phenotypes [120,121,122]. An analysis of sewage samples from 79 cities across 60 countries reveals that phage-borne ARGs can be transferred among diverse hosts, which suggests that phages may play a pivotal role in the global dissemination of ARGs [8]. Additional studies confirm that transduction mediated by bacteriophages can facilitate the movement of ARGs between bacteria to enhance resistance levels within microbial populations [123]. This phage-driven genetic exchange is particularly effective in environments characterized by high microbial density where host–virus interactions are more frequent [124]. However, lytic phages such as BVPaP-3 exhibit high host specificity and only lyse specific bacterial strains [125]. This primarily restricts the transduction of antibiotic resistance to bacteria of the same species or those that are closely related phylogenetically [124]. Moreover, exposure to sub-lethal concentrations of antibiotics in complex environmental contexts can trigger prophage induction, increase the frequency of lytic cycles, and promote ARG transfer through transduction [123].

## 8. Conclusions and Perspective

Soil viruses regulate microbial life and nutrient turnover. In recent years, soil virology has transitioned from a descriptive phase to a stage focused on elucidating functional mechanisms owing to the improvement of multi-omics technologies. Soil viruses represent the Earth’s largest reservoir of unknown genes and play irreplaceable roles in ecosystems. They mediate host community structures via lytic strategies, accelerate the release of intracellular nutrients, and participate in biogeochemical cycles using AMGs. Although research in this field is rapidly expanding, soil viral ecology still faces numerous challenges. Future research should focus on the three aspects described below.

- Methodological improvement and database expansion. This is essential to optimize extraction and purification methods for soil viruses and establish standardized protocols. Furthermore, databases including largely overlooked RNA viruses and eukaryotic viruses (such as those that infect fungi and plants) are warranted.
- Multi-scale distribution patterns of viruses. The distribution patterns of viruses across multiple ecosystems can reveal the relationships between climate change, land-use practices, and the dynamic changes in soil viral communities.
- In situ interaction mechanisms and model integration. A deeper understanding of “virus–host” in situ interactions within the complex soil matrix is required, particularly regarding the spatiotemporal dynamics of temperate phages. More importantly, processes mediated by viruses—such as lysis, gene transfer, and nutrient cycling—need to be incorporated into macroscopic biogeochemical and ecosystem predictive models.

## Figures and Tables

**Figure 1:**
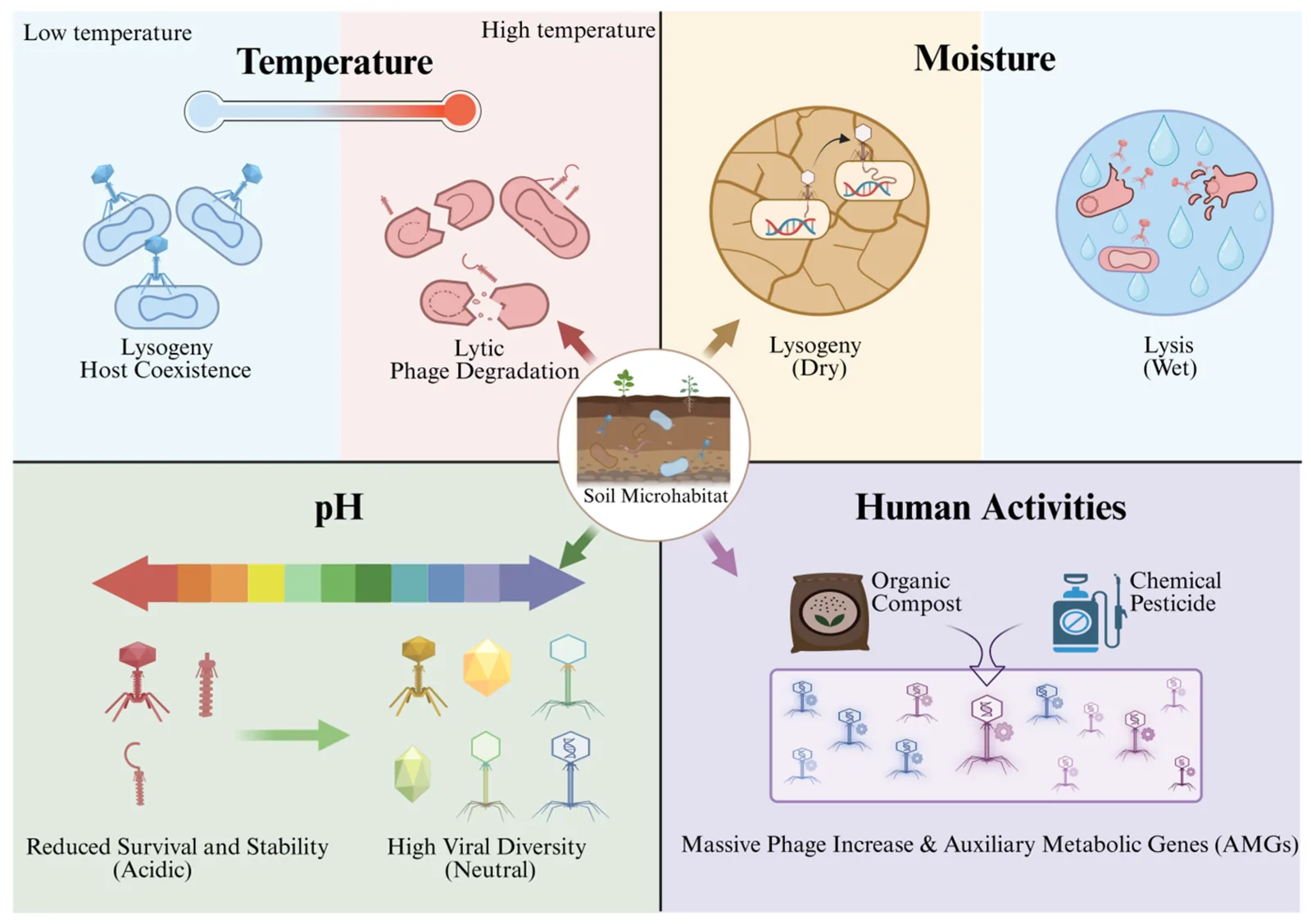
Effects of environmental factors and human activities on the survival, diversity, and life cycle of viruses in soil. Created in BioRender. Fantong, S. (2026) https://BioRender.com/u3v4jr3.

**Figure 2:**
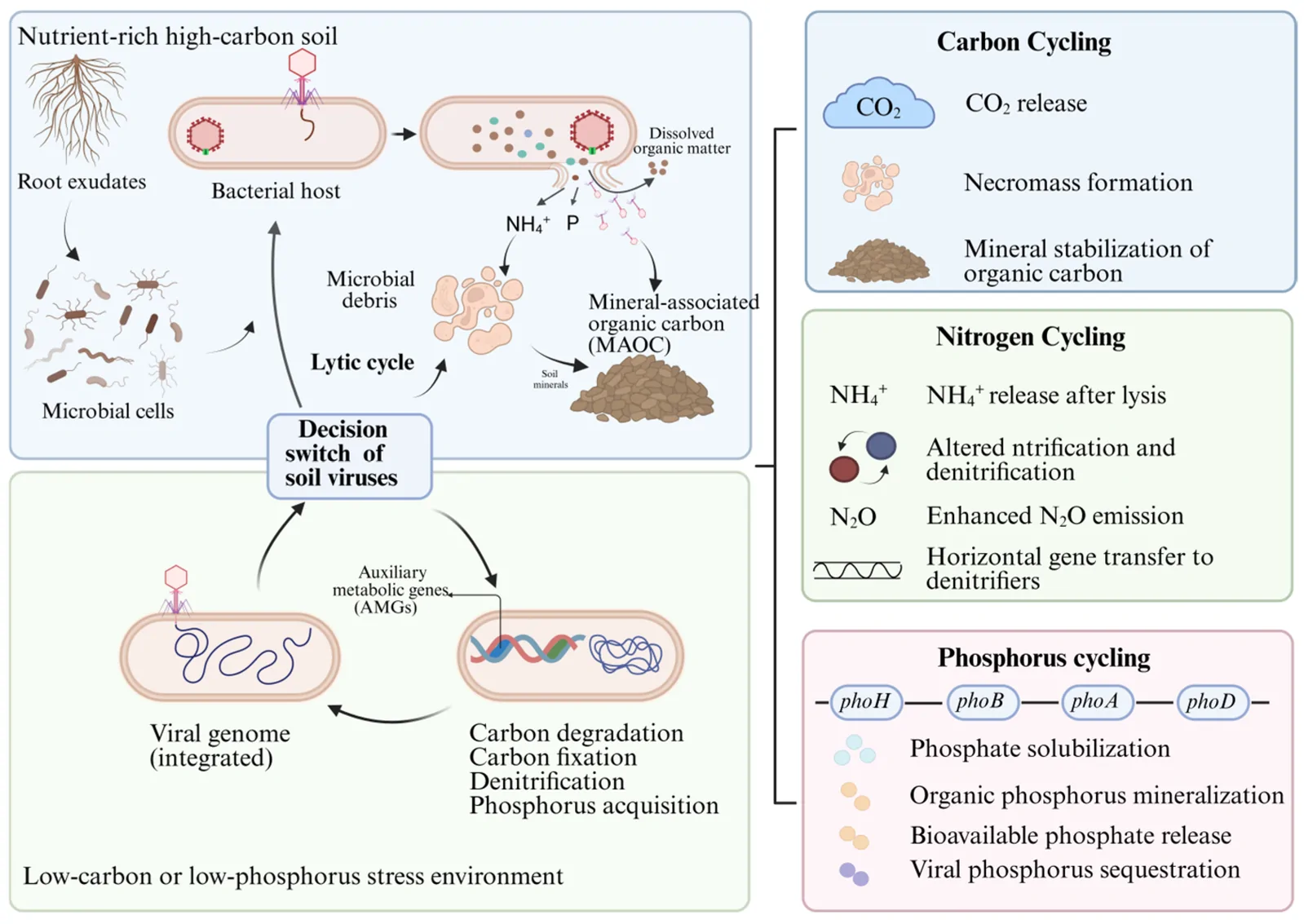
Roles of soil viruses in mediating host interactions and regulating key biogeochemical cycles in ecosystems. Created in BioRender. Fantong, S. (2026) https://BioRender.com/tpe52yb.

**Figure 3:**
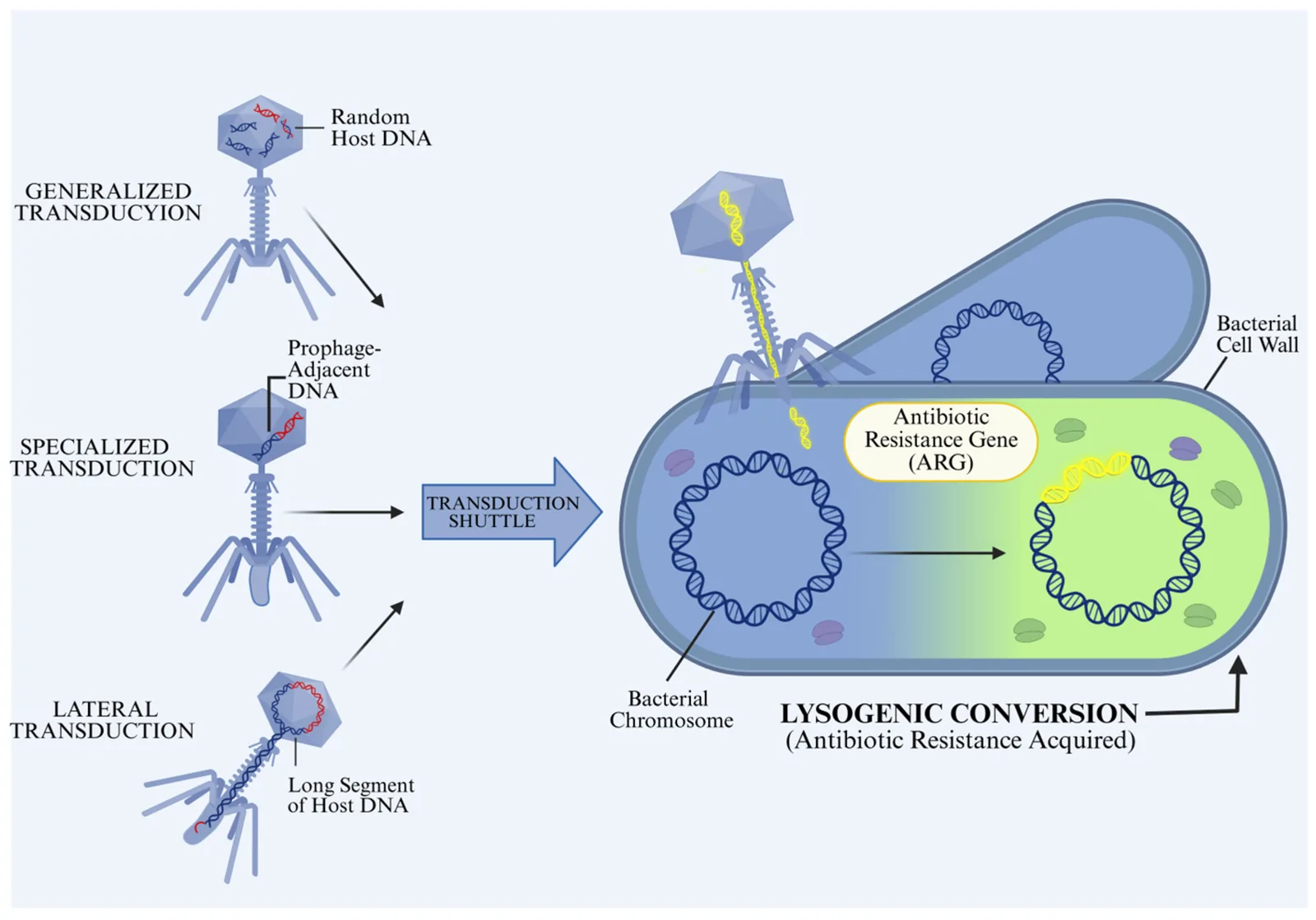
Schematic illustration of the transduction shuttle process in bacteria. Created in BioRender. Fantong, S. (2026) https://BioRender.com/j80vsrb.

## Data Availability

No new data were created or analyzed in this study. Data sharing is not applicable to this article.
